# Crude microcystins extracted from *Microcystis aeruginosa* exert anti-obesity effects by downregulating angiogenesis and adipogenesis related signaling molecules in HUVEC and 3 T3-L1 cells

**DOI:** 10.1186/s12906-019-2501-0

**Published:** 2019-05-08

**Authors:** Muhammad Imran Khan, Jin Hyuk Shin, Jong Deog Kim

**Affiliations:** 1 Department of Biotechnology, Chonnam National University, San96-1, Dun-Duk Dong, Yeosu, Chonnam 550-749 South Korea; 2 Research Center on Anti-Obesity and Health Care, Chonnam National University, San96-1, Dun-Duk Dong, Yeosu, Chonnam 550-749 South Korea

**Keywords:** Angiogenesis, Adipogenesis, Microcystin, Obesity, Oil red O staining, VEGFR-2

## Abstract

**Background:**

Obesity is a risk factor for many diseases including diabetes, cancer, arthritis, and cardiovascular diseases. Angiogenesis nourishes adipose tissues and contributes to obesity; it can be prevented by suppressing the expression of associated signaling molecules. Natural products have garnered attention owing to their safety and efficacy in treating several diseases, including obesity.

**Methods:**

Crude Microcystins were extracted from the blooming *Microcystis aeruginosa* under stress conditions, by ultrasonication following by solvent extraction. The microcystin extract was evaluated for its potential of inhibiting angiogenesis and adipogenesis. The antiangiogenic activity of the microcystins extract was investigated using human umbilical vein endothelial cells (HUVECs), and its anti-obesity activity was determined in vitro by quantification of the accumulated lipids in mouse 3 T3-L1 cells via Oil Red O staining method.

**Results:**

The microcystin extract suppressed HUVECs proliferation and tubes formation in Matrigel in a dose-dependent manner. RT-PCR analysis revealed the downregulation of the mRNA expression of angiogenesis-related signaling molecules, such as PI3K, β-catenin, vascular endothelial growth factor receptor-2 (VEGFR-2), vascular endothelial-cadherin, Akt1, and NF-κB. Additionally, it inhibited the differentiation of premature 3 T3 cells and lipid accumulation in a dose-dependent manner. It suppressed adipogenesis and lipogenesis by reducing the expression level of peroxisome proliferator-activated receptor γ, CCAAT/enhancer binding protein α, and sterol regulatory element-binding protein.

**Conclusions:**

Crude microcystin exerts anti-angiogenic and anti-obesity effects due to the inhibitory effects on the genes expression of associated signaling molecules and transcriptional factors.

## Background

Angiogenesis is a physiological process that involves the development of new blood vessels from pre-existing ones; it is crucial for uterus functioning, embryogenesis, and wound healing [1]. It breaks cell-cell contacts and degrades the endothelium and extracellular matrix to facilitate the proliferation and migration of endothelial cells and formation of capillary tubes [1, 2]. Although angiogenesis is essential for normal physiological functioning, it can drive the pathogenesis of certain diseases such as cancer, atherosclerosis, arthritis, diabetic retinopathy, and ischemic stroke [3, 4]. By supplying the tumors with nutrients, angiogenesis promotes tumor growth and progression [5, 6]. Hence, angiogenesis causes tumor growth and expansion. Several activators and inhibitors are involved in the regulation of angiogenesis. The major angiogenic signaling proteins are vascular endothelial growth factor (VEGF), platelet-derived growth factor, angiopoetin-1/2, interleukin-8, basic fibroblast growth factor, and angiotensin II [7–15]. Angiogenesis can be inhibited by suppressing the activity of VEGF, which plays a key function in endothelial cell proliferation and migration. Signaling molecules that regulate angiogenesis function in an interconnected complex network of biochemical pathways. Inhibition of any component of this network will lead to the inhibition of angiogenesis.

Obesity is one of the leading causes of death worldwide, accounting for 2.8 million deaths annually. According to a WHO report in 2008, four billion people were overweight, and 500 million individuals among them were obese. Obesity increases the risk of cardiovascular diseases, ischemic heart disease, diabetes, and various cancers [16]. Adipocytes are surrounded by an extensive network of capillaries that supplies oxygen, hormones, and cytokines, thereby promoting their proliferation, growth, and differentiation. This vascular network crucially regulates adipogenesis [17, 18]. Because angiogenesis plays a key role in the growth and proliferation of adipocytes, its inhibition holds potential to treat obesity and metabolic disorders. The differentiation of mature adipocytes from pre-adipocytes involves numerous transcription factors [19]. This process is characterized by morphological changes in the adipocytes. Transcriptional factors, such as peroxisome proliferator-activated receptor γ (PPARγ) and CCAAT/enhancer binding protein α (C/EBPα), regulate adipogenesis [20]. The suppression of PPARγ and/or C/EBPα inhibits adipogenesis [21].

Currently, different types of synthetic anti-obesity agents are available commercially. Despite their pharmacological benefits, they are reported to cause adverse effects; these pharmaceuticals not only inhibit adipocytes, but also damage normal cells [22].

Natural products have garnered interest owing to their safety and efficacy in treating obesity [23–25]. They offer the safest and most economical alternatives for the treatment of various diseases, such as cancer, diabetes, and obesity. Microalgae have started gaining popularity because of the abundant bioactive molecules present in them. Algae are cultured as a source of food, biofuels, and biopharmaceuticals. Moreover, microalgae and cyanobacteria hold profound medicinal and nutritional significance; their extracts are used in cosmetics, medicinal products and nutraceuticals [26–28]. Biotechnological processing of natural substances from algae and cyanobacteria has recently gained popularity. They contain a variety of valuable compounds finding wide applicability. It is estimated that approximately 5000 t of dry algal biomass used in bioproduction generates USD 1.25 billion each year [29]. Serval important species of microalgae have been identified. *Microcystis aeruginosa* is a freshwater photosynthetic microalga (cyanobacterium). That form algal bloom on the surface of water bodies. Such algal blooms are observed in fresh water rivers and lakes in many regions of the world. During blooming, *M. aeruginosa* releases toxic metabolites called microcystins (cyanoginosins). Microcystins are cyclic peptides, and most of them are Adda peptides [30, 31]. Approximately 90 types of microcystins have been reported [32]. This toxic metabolite of *M. aeruginosa* has been reported to possess cytotoxic and antimicrobial activities. In this study, we investigated the anti-angiogenic and anti-obesity effects of crude microcystins isolated from *M. aeruginosa* with the underlying mechanisms.

## Methods

### *M. aeruginosa* culture preparation and extraction of microcystins

*Microcystis aeruginosa* (KMMCC-1135) used in this study was obtained from the Korea Marine Microalgae Culture Center (KMMCC) and was cultured under stress conditions in MF media [33] supplemented with NaCl. When the culture attained log phase growth (OD_660_ = 1), it was subjected to high temperature(35°C for one week ) and then dewatered to yield a concentrated culture (OD_660_ = 1.8). The concentarted culture was subjected to UV radiation for 4 h daily for one week. Further, the culture was lyophilized, washed twice with PBS buffer, and subjected to ultrasonication for 5 min.

After disruption of the cells, the sonicated algal hydrolysate was used for microcystin extraction following the method of Oudra et al. 2001 [34]. Butanol, methanol and water (1:4:15) was used for the extraction of the crude microcystins from the hydrolysate. Extraction was continiued for 1 h, after which the solution was centrifuged for 25 min at 20,000×*g*. The pellet obtained was subjected to overnight extraction using the same solvent system. After centrifugation, the supernatant was collected and allowed to evaporate to about 30% of the total volume to yield crude microcystins (500 μL). The microcystin extract was sterilized by syringe filtration and stored at − 20 °C until further analysis. Aqueous Solution of the microcystin was then used for antiangiogenic and anti-obesity assays.

All the cell lines (HUVECs and 3 T3-L1) used in this study were obtained from American Type Culture Collection (ATCC).

### Antiangiogenic activity analysis

#### Cell viability assay

Cell viability was determined by the MTT assay. Human umbilical vein endothelial cells (HUVECs) were used for this assay. They were cultured in an endothelial growth basal medium-2 (Clonetics, USA) supplemented with an endothelial cell growth medium-2 (Clonetics) in a 96-well plate (1 × 10^4^ cells/well) at 37 °C in a 5% CO2 humidified incubator. After 24 h (80% confluency), the cells were treated with various concentrations of the crude microcystin for 24 h at 37 °C in a humidified 5% CO_2_ incubator. MTT (0.5% *w*/*v*) was added to the medium in each well of the plate, and the plate was incubated for 4 h. After aspirating the medium from each well, 200 μL of DMSO was added to dissolve the formazan crystals, and the cells were incubated for 15 min. Absorbance was read at 590 nm with a reference filter of 620 nm on a microplate reader (Biochrom Ltd.) and percentage cell viability was calculated. Cells not treated with the sample (microcystins) were taken as negative control.

#### In vitro antiangiogenic activity

The antiangiogenic activity of the isolated microcystin extract was determined by its inhibitory effects on tubular structures formation by HUVECs in Matrigel (BD Bioscience, MA, USA). Matrigel was added to 24-well plates and was allowed to solidify at 37 °C. HUVECs were seeded in the Matrigel-coated wells (2.5 × 10^4^ cells/well), and the plates were incubated at 37 °C for 4 h. The cells in the well were treated with various concentrations of the microcystin extract (5-60 μl/mL). 50 μl of each concentration was added to the respective wells and the plates were incubated for 4–5 h. Tube formation by the HUVECs was observed under a phase contrast inverted microscope (Nikon, Tokyo, Japan); five random sides of each well were photographed and analyzed using the Scion Image software (NIH, ML, USA). Wells containing untreated cells were considered as control.

#### Effects of microcystins on the genes expression of angiogenesis promoting signalling molecules

To determine the effect of the microcystin extract on the expression of angiogenic proteins, RT- PCR analysis was conducted using gene-specific primers. HUVECs were cultured in an endothelial growth basal medium-2 supplemented with 0.3% FBS in a 6-well plate. The cells were then incubated with different concentrations of the microcystin extract (5–60 μl/mL) for 24 h. Total RNA was isolated from the HUVECs, using the TRI reagent (Sigma Aldrich). cDNA was synthesized from the RNA, using the Revert Aid First Strand cDNA Synthesis Kit. Gene-specific primers were used for quantification of gene transcripts for VEGFR, PI3K, VE-cadherin, β-catenin, AKT, NF-kB and β-actin by real-time PCR using the following conditions; 94 °C or 5 min, followed by 35 three-step cycles including denaturation at 94 °C for 1 min, annealing at 58 °C for 1 min, extension at 72 °C for 1 min followed by final extension at 72 °C for 5 min. The products were analyzed by gel electrophoresis.

The primers used were, VEGFR, forward, 5ʹ−AGG TTG CGT GTT CTT CGA GT-3ʹ and reverse, 5ʹ−CCC AAA GTG CTG GGT TTT TA-3ʹ; PI3K, forward, 5ʹ−CGT GTG CCA TTT GTT TTG AC-3ʹ and reverse 5ʹ−TCA AAC CCT GTT TGC GTT TAC-3ʹ; VE-cadherin, forward, 5ʹ−GGA AGG AGA CAC CAA GCT CA-3ʹ and reverse 5ʹ−CTT GTC ATG CAC CAG TTT GG-3ʹ; β-catenin, forward, 5ʹ−GGT GGG CTG GTA TCT CAG AA-3ʹ and reverse, 5ʹ− GGC AAC TGG TAA ACT GTC CAA-3ʹ; AKT, forward, 5ʹ−CCG ATT CAC GTA GGG AAA TG-3ʹ and reverse, 5ʹ−AGC GTC GAA AAG GTC AAG TG-3ʹ; NF-kB forward, 5ʹ−TGG TCA GCT CCC TTC TCT GT-3ʹ and reverse, 5ʹ−GCC AGC TTG GCA ACA GAT-3ʹ; β-actin, forward, 5ʹ−CTC CTG AGC GCA AGT ACT CC-3ʹ and reverse, 5ʹ−ACA TCT CAA GTT GGG GGA CA-3ʹ.

#### Anti-obesity activity analysis

The anti-obesity effect of crude microcystins was determined by examining their inhibition of 3 T3-L1 cell proliferation. The cells were cultured in DMEM (Gibco, Grand Island, NY, USA) containing 100,000 IU/L penicillin, NaHCO_3_ (3.7 g/L), 10% (*v*/v) FBS, and 100 mg/L streptomycin.

#### Cell viability assay

Cell viability was determined by the MTT assay (Methylthiazolyl tetrazolium assay). Mouse preadepocytes 3 T3-L1 cells were cultured in a 96-well plate (1 × 10^4^ cells/well) in a humidified 5% CO_2_ incubator for 24 h at 37 °C, after which they were treated with different concentrations of the microcystin extract for 24 h. Cells not treated with microcystin were taken as negative control. Further, MTT (3-(4,5-dimethylthiazol-2-yl)-2,5-diphenyltetrazolium bromide) (0.5% *w*/*v*) was added to each well, and the plate was incubated for 4 h. After aspirating the medium from each well, DMSO (Dimethyl sulfoxide) (Sigma Aldrich, MO, USA) was added for dissolving the formazan crystals. Absorbance was read at 540 nm using a microplate reader (Biochrom Ltd., UK). Cell viability was calculated as a percentage relative to control.

### Determination of the anti-obesity activity

3 T3-L1 cells (1 × 10^4^ cells/well) were cultured in 6-well plates. After two days (80% confluency), the culture medium was replaced with a differentiation medium containing insulin (10 μg/mL), dexamethasone (1 mM), and isobutyl-1-methylxanthine (0.5 mM), and the cells were cultured in this medium for 24 h to arrest cell division. After two days, the differentiation medium was replaced with a maintenance medium containing DMEM supplemented with insulin (10 μg/mL). The medium was replaced every two days up to eight days. The microcystin was added at different concentrations (5–60 μl/mL) during the addition of the differentiation and maintenance media. Epigallocatechin gallate (50 μg/mL) was used as positive control and cells not treated with microcystins were taken as negative control. After eight days of maintenance culture, the cells were washed with PBS and then fixed with formalin (10%) for 1 h. Further, the cells were washed with 60% isopropanol and stained with the Oil Red O solution. The cells were then incubated at 37 °C for 3 h. They were washed four times with distilled water and again with isopropanol for extracting the staining dye. The absorbance of the extracted Oil Red O solution was then measured at 520 nm on a spectrophotometer.

### Genes expression level analysis of the adpogenesis and lipogenesis promoting signaling proteins 

To study the effect of the microcystin extract on the expression of adipogenesis- and lipogenesis-related transcriptional factors, such as PPARγ, C/EBPα, and sterol regulatory element-binding protein (SREBP), premature 3 T3 cells were cultured in T75 flasks in DMEM and incubated in a CO_2_ incubator up to 70% confluency. The cells were then treated with the safe concentrations of the algal crude microcystins (5–60 μL) and the cells without samples (non-treated) were taken as negative control. After 24 h, RNA was isolated from the cells by treating with the TRI reagent (Sigma Aldrich). The isolated RNA of each sample was reverse-transcribed to cDNA, using Revert Aid First Strand cDNA Synthesis Kit (Thermo Fisher Scientific). The cDNA was converted to DNA by PCR. Gene-specific primers were used for quantification of gene transcripts for C/EBPα, SREBP and PPARγ, by real-time PCR using the following conditions; 94 °C or 5 min, followed by 35 three-step cycles including denaturation at 94 °C for 1 min, annealing at 60 °C for 1 min, extension at 72 °C for 1 min followed by final extension at 72 °C for 5 min. The amplified products were analyzed by agarose gel electrophoresis.

The primers used were C/EBPα- forward, 5ʹ− TTA CAA CAG GCC AGG TTT CC -3ʹ and reverse, 5ʹ− CCA CAG GGG TGT GTG TAT GA-3ʹ; SREBP, forward, 5ʹ− TTG CAC CAG AGA GCA TTT TG-3ʹ and reverse 5ʹ− GAA AAT GAG AGG CTG GTT GC-3ʹ; PPARγ, forward, 5ʹ− CTG GCC TCC CTG ATG AAT AA -3ʹ and reverse 5ʹ−CTT GTC ATG CAC CAG TTT GG-3ʹ; β-actin, forward, 5ʹ−CTC CTG AGC GCA AGT ACT CC-3ʹ and reverse, 5ʹ−ACA TCT CAA GTT GGG GGA CA-3ʹ.

### Statistical analysis

Statistical analyses were performed by Analysis of variance (ANOVA) with post-hoc comparison by Tukey’s test at α = 0.05. Normal distributions of the data in groups were first analyzed before using ANOVA. Normality was determined by Kolmogorov-Smirnov (K-S) test. Data are approximately normally distributed with *p* = 0.162, at the 0.05 level of significance. Homogeneity of variances in groups were determined by Levene’s test. Levene’s test was not significant and variances in the groups were nearly same at Sig. 0.176. All experiments were performed at least in triplicates and means were taken and data represented as the mean ± SEM. Data are statistically significant at (*P* < 0.05). SPSS was used as a statistical program.

## Results

### Antiangiogenic activity

**Fig. 1:**
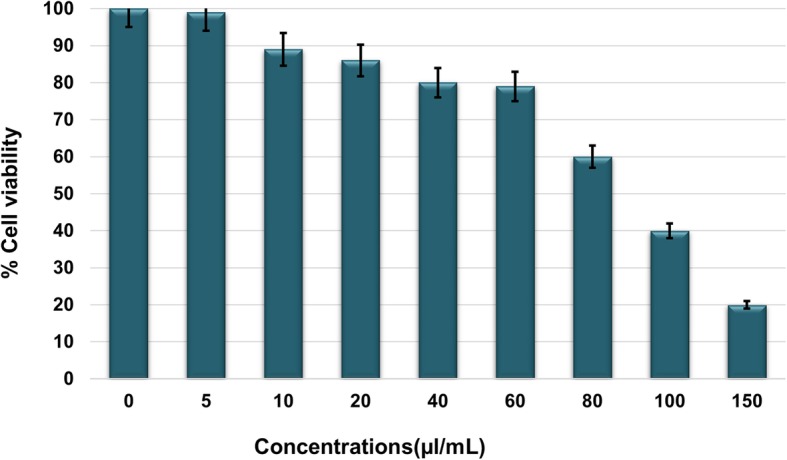
Cell viability determination by MTT assay. Viability of HUVECs under the treatment of various concentrations of the crude microsystems. Data are expressed as means of experiments in triplicate ± SEM. Data are statistically significant at *P* < 0.05

The toxicity of different doses of the microcystin extract on HUVECs was determined by the MTT assay. Figure 1 represents cell viability assay for HUVECs. This assay was conducted to determine the concentrations range of microcystins which is not toxic to cells and maintain about 80% cells viability. Various concentrations of microcystin extract i.e. 5, 10, 20, 40,60, 80, 100 and 150 μl/mL were used, and it was found that the cells remain viable (more than 80%) under microcystisen concentrations up to 60 μl/mL beyond this concentration cells viability declined as shown in Fig. 1. Hence 60 μl/mL were determined as maximum safe concentration and for exploring antiangiogenic potential of the crude microcystisen, concentrations of 5, 10, 20, 40 and 60 μl/mL were used.

**Fig. 2:**
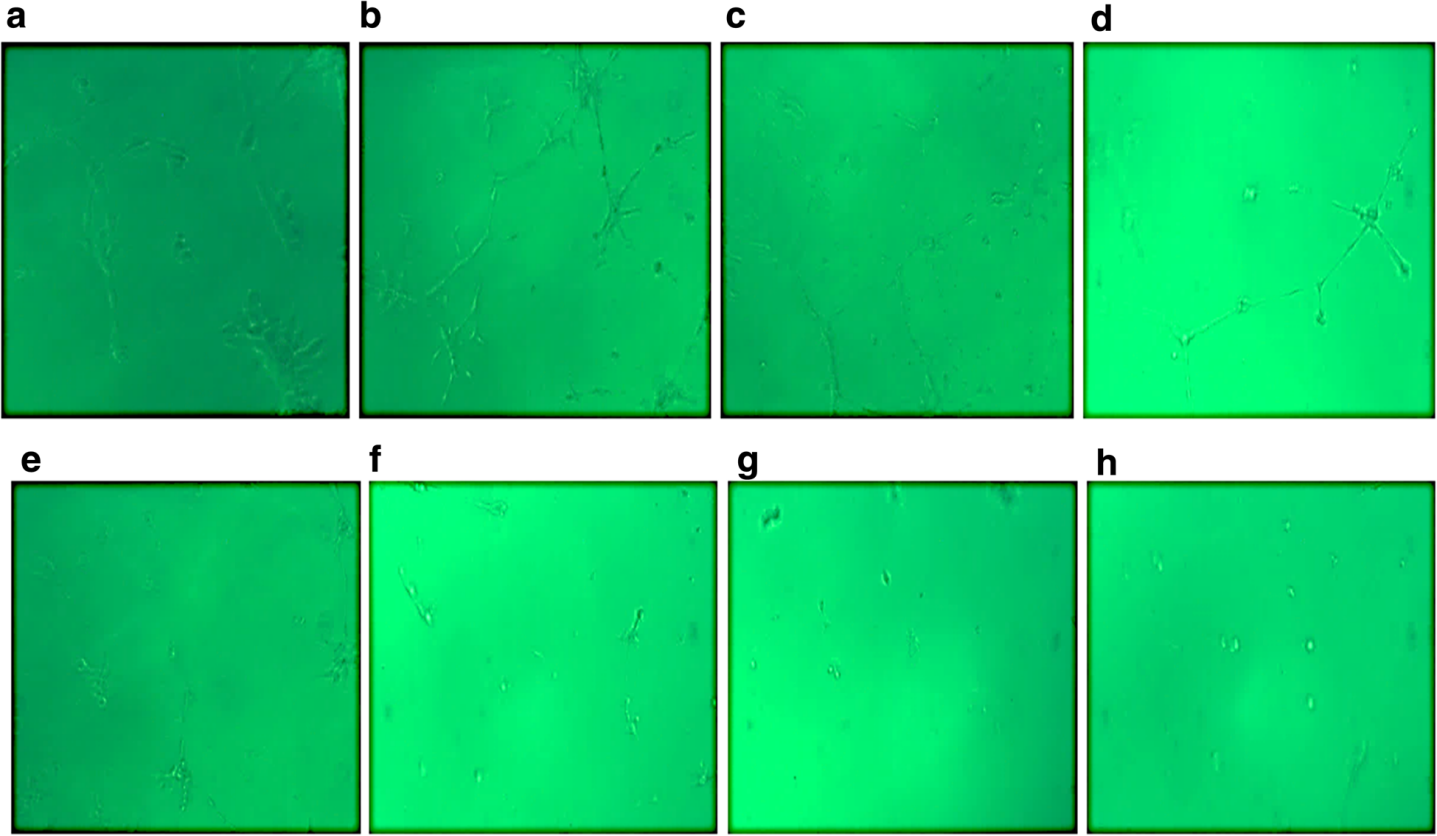
Effects of crude microsytins on HUVECs tubes formation in Martigel. Various concentrations (5, 10, 20, 40 and 60 μl/mL) of microcystin were used to study its inhibitory potential on HUVECs proliferation in Matrigel. HUVECS were seeded in 96 well microtiter plate and incubate for 24 h under various concentrations of crude microcystin (control without microcystins) and photographed randomly at three different sites. **a, b** represents the photographs of HUVECs in control well (HUVECs without treatment with microcystins). **c, d** represents the HUVECs photographs shot after treatment with the lowest concentration i.e. **5** μl**/mL** of microcystin extract. **e, f, g and h** represent the photograph of HUVECs in different wells treated with different concentration of crude microcystin in the range of **10, 20, 40 and 60 μl/mL** respectively

**Fig. 3:**
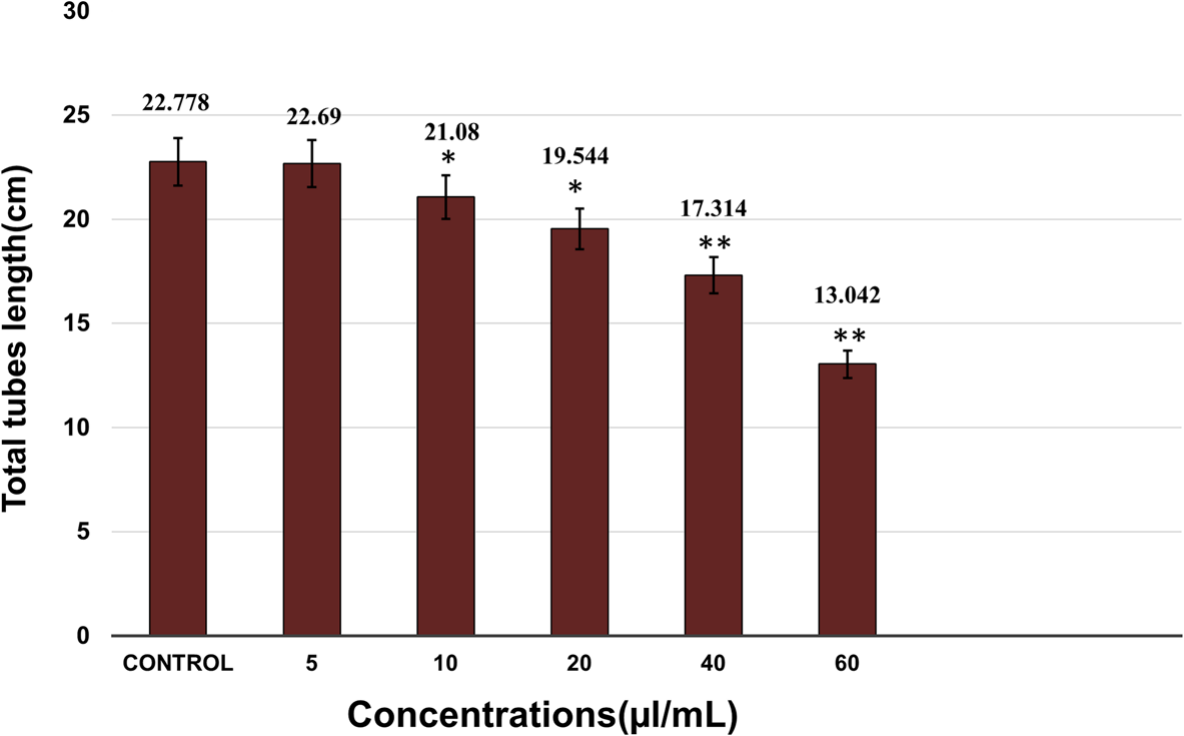
Inhibitory effects of crude microcystins on HUVECS tubular structures formation at various concentrations (5–60 μl/mL). Total tubes lengths of HUVECs on Matrigel in the presence of various concentrations of microcystins. Data are expressed as means of values ± SEM. * *p* < 0.05 and ** *p* < 0.01 as compared to control

The in vitro inhibition of HUVEC proliferation in this dose range was then evaluated by the Matrigel assay. The antiangiogenic activity of the crude microcystins was determined by the inhibitory effects on the proliferation of HUVECs i.e. the inhibition of the tubes formation by HUVECs in Matrigel. From the results it was found that microcystins suppressed tube formation by HUVECs in a dose-dependent manner. Figure 2 Shows the  photographs of HUVECs treaded with crude microcystin shot with phase contrast inverted microscope (Nikon, Tokyo, Japan) connected to a camera. Tottal tubes length were calculated for each group from the photographs analysed with Scion Image software (NIH, ML, USA). Total lengths of tubes formed by microcystin-treated HUVECs were compared with those formed by control (untreated HUVECs). Tube lengths were found to decrease with increasing concentrations of the crude microcystins (5 to 60 μl/mL) which indicates the effectiveness of the microcystin against HUVECs proliferation (Fig. 3). These results showed that the extract inhibits tube formation by HUVECs as well as their proliferation in a dose-dependent manner, verifying its antiangiogenic activity.

### Genes expression analysis of angiogenesis promoting sinaling proteins 

Because the crude microcystin inhibited tube formation by HUVECs, we conducted RT-PCR analysis to conform the results and evaluate the mechanisms of action. The expression of angiogenesis-promoting signaling molecules, such as PI3K, β-catenin, VEGF receptor (VEGFR)-2, and VE-cadherin, ware evaluated using gene-specific primers as described in methods section.

**Fig. 4:**
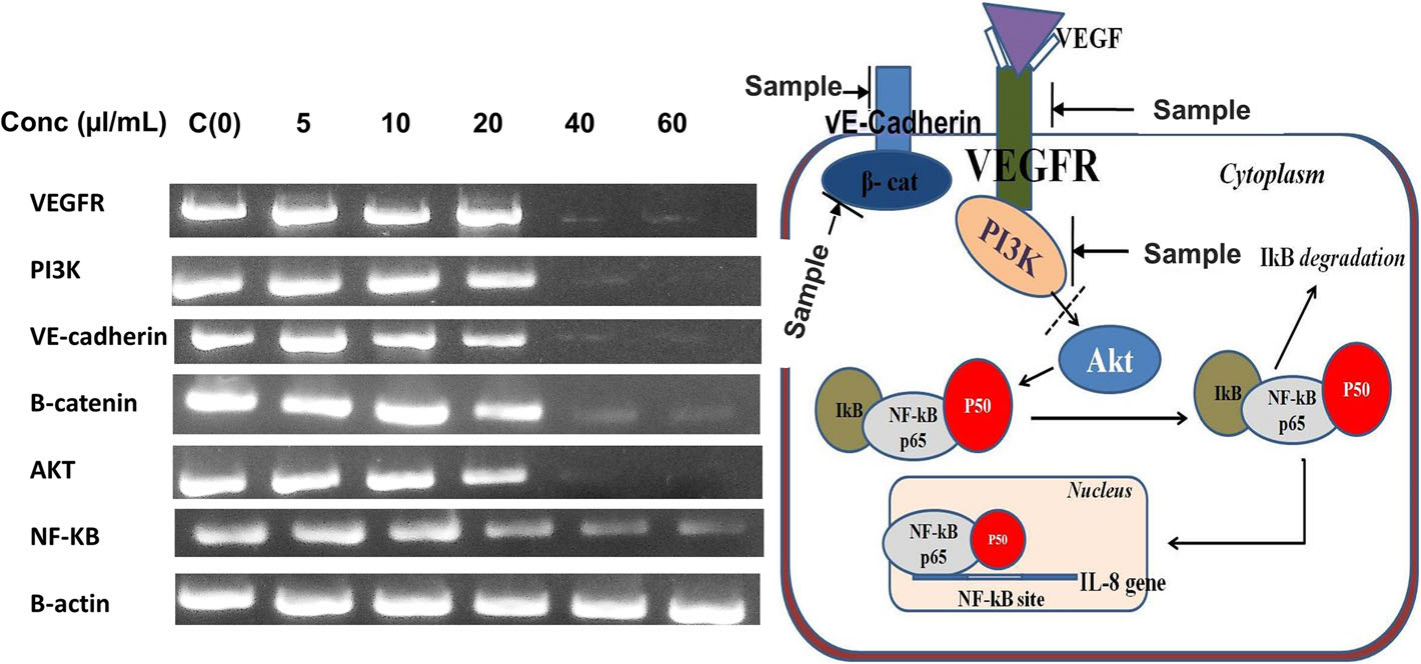
Inhibitory effects of crude microcystins on mRNA expression level of VEGFR-2, PI3 K, β-Catenin, VE-Cadherin, Akt, and NF-kB analysed by RT-PCR **b.** The possible mechanisms of the inhibition of angiogenesis by the crude microcystins. We supposed and graphically described the mechanism of inhibition of angiogenesis by crude microcystin. 500 μl microsytins were isolated from 10 ml *M. aeruginosa* culture (OD660 = 1.8) and then different volumes of the crude microcystins were (5–60 μl/mL) used for the antiangiogenic assay

The microcystin extract reduced the expression of these signaling molecules in a dose-dependent manner. It also reduced the expression of Akt1 and NF-κB, thereby inhibiting angiogenesis (Fig. 4a). The possible mechanisms of its antiangiogenic actions are shown in Fig. 4b.

**Fig. 5:**
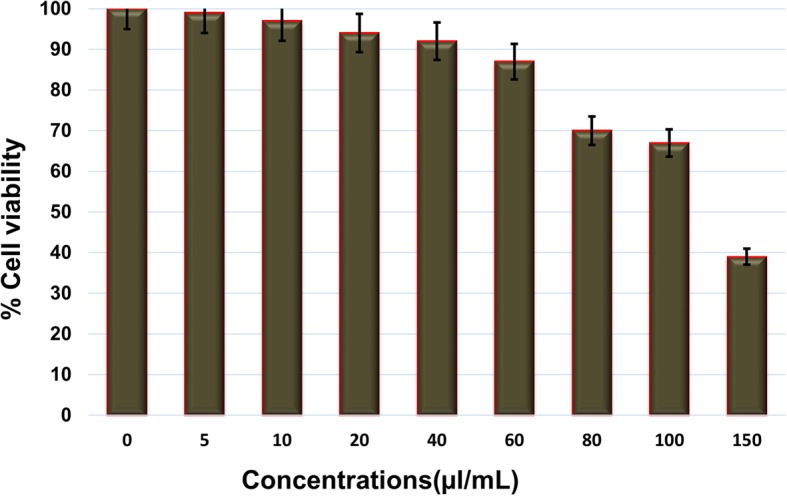
Safe and toxic dose determination of crude microcystins for 3 T3 cells by MTT assay. Viability of 3 T3-L1 cells under the treatment of various concentrations of microcystins. Data are expressed as means of values ± SEM. Data are statistically significant at *P* < 0.05

### Anti-obesity activity

**Fig. 6:**
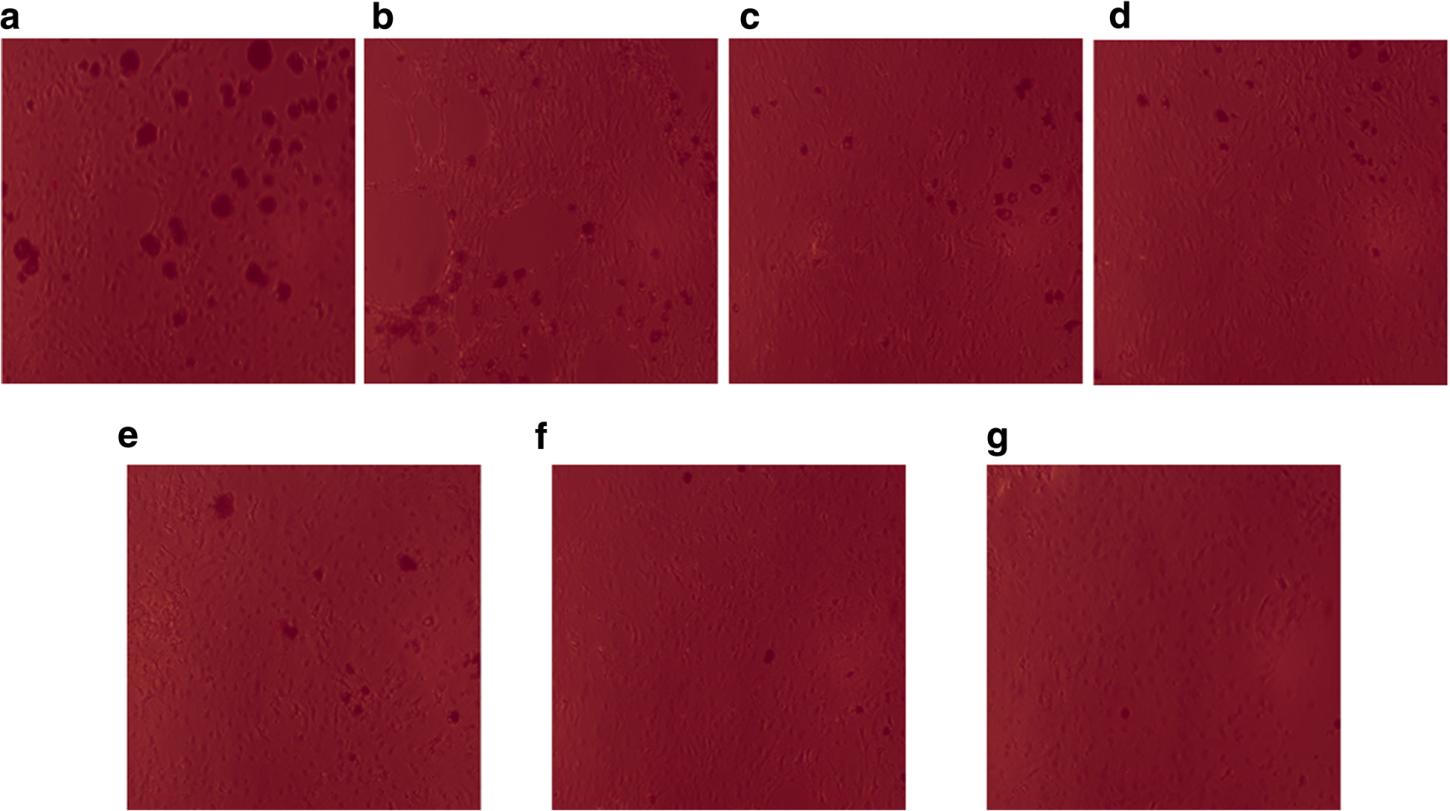
The inhibition of 3 T3 cells differencing and lipid accumulation by various concentrations of crude microcystins determined by Oil Red O staining. **a** represent the photograph of 3 T3-L1 cells in control well (**0 μl/mL** microcystins). **b, c** represents the photographs of lowest dose group of microcystins extract i.e. **5 μl/mL**. **d, e, f and g** represent the photograph of 3 T3-L1 cells line after treatment with crude microcystin in the concentration range of **10, 20, 40 and 60 μl/mL** respectively

**Fig. 7:**
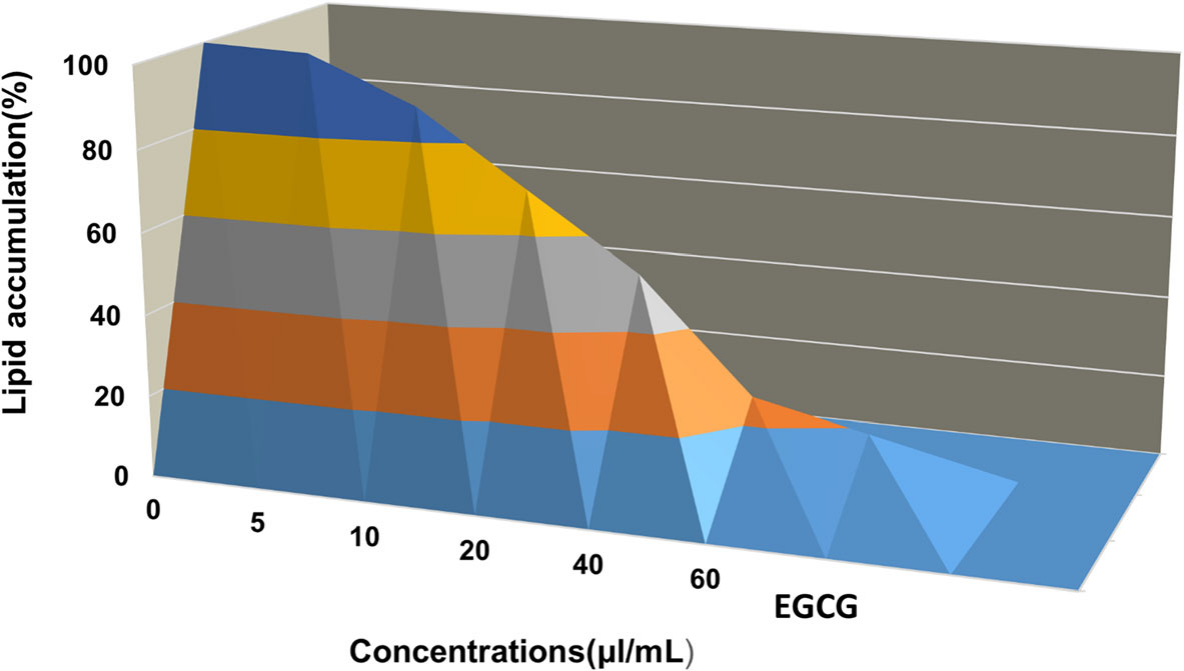
Inhibition of 3T3-L1 differentiaition and lipid accumulation by various concentrations of crude microcystins (control without microcystins) from the lowest concentration 5 μl/mL to the highest concentration respectively 60 μl/mL). Data are expressed as means of values ± SEM. Data are statistically significant at *P* < 0.05

The in vitro anti-obesity activity of the microcystin extract was determined by observing the proliferation and differentiation of 3 T3 premature adipocytes in the presence of various concentrations of the microcystin extract. Cell viability was determined by the MTT assay, and the safe dose range of microcystins extract was determined. The viability of 3 T3-L1 cell line under microcystins was found nearly similar to that of HUVECs i.e. 60 μL/mL was found to be the maximum safe concentration (Fig. 5). Hence the microcystins extract were used in the concentrations range of 5,10, 20, 40 and 60 μL/mL as nontoxic doses for anti-obesity activities. EGCG was used as the positive control (50 μg/mL). The microcystin extract inhibited lipid accumulation by inhibiting the proliferation and differentiation of 3 T3 adipocytes. Results of the Oil Red O staining assay showed that the extract inhibited lipid accumulation in a dose-dependent manner. Figure 6 represents the accumulated lipidis in 3 T3-L1 cells proliferated in the pressence(5,10, 20,40 and 60 μL/mL) or absence(control) of crude microcystins photographed with phase contrast inverted microscope (Nikon, Tokyo, Japan) connected with a camera. The absorbance (520 nm) of the extracted Oil Red O solution after extraction from the 3 T3-L1 adipocytes was found to be decreased with the increasing concentrations of microcystins (Fig. 6). These results indicated that the microcystin extract inhibits lipid accumulation in 3 T3-L1 adipocytes; at the highest safe concentration of 60 μL/mL, the extract suppressed lipid accumulation almost as effectively as EGCG shown in Fig. 7.

### Analysis of adipogenesis- and lipogenesis-related genes expression level

**Fig. 8:**
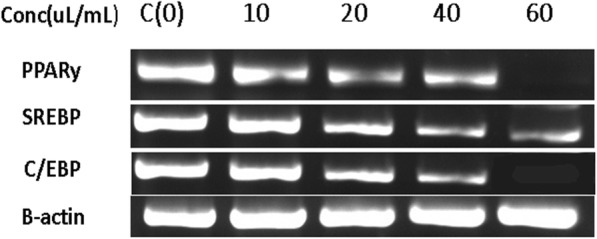
Expression levels of PPARy, SREBP and C/EBPα, in the RNA extracted from 3 T3 cells treated with various concentrations of microcystins, analyzed by RT-PCR. Different concentrations of the microcystins (5–60 μl/mL) were used to determine the inhibitory effects of crude microcystins on the gene expression levels of the adipogenesis promoting signaling molecules

To identify the mechanisms by which the microcystin extract inhibits the differentiation and proliferation of premature adipocytes, we analyzed the mRNA expression of adipogenesis- and lipogenesis-related signaling molecules, such as PPARγ, C/EBPα, and SREBP, by RT-PCR using gene-specific primers as describes in methods section. The microcystin extract inhibited lipogenesis and adipogenesis in 3 T3 premature adipocytes by suppressing the expression of these signaling molecules in a dose-dependent manner (Fig. 8).

## Discussion

Angiogenesis assists tumor progression and metastasis [35]. Obesity also develops through a mechanism similar to that responsible for angiogenesis [36]. VEGF predominantly regulates angiogenesis by activating downstream signaling molecules such as VEGFR-2, PI3K, β-catenin, VE-cadherin, and Akt1. This further promotes NF-κB-mediated Bcl-2 activation and IL-8 expression to induce cell proliferation and angiogenesis [37–40]. VEGF also crucially regulates adipocyte differentiation [41]. In the present study, we evaluated the potential of crude microcystins to inhibit angiogenesis in vitro in HUVECs. Our results confirmed that the microcystin extract derived from *M. aeruginosa* suppresses angiogenesis by inhibiting endothelial cell proliferation and tubes formations. Our results show similarity with the findings of Shi et al. 2017 [42] who reported that MC-LR (40 μM) decreased cell migration and tube formation of HUVECs. RT-PCR analysis showed that it suppresses expression of key signaling molecules related to angiogenesis (VEGFR-2, PI3K, β-catenin, VE-cadherin, Akt1, and NF-κB) in a dose-dependent manner.

Obesity is emerging as a serious health concern. It is a metabolic disorder characterized by the expansion of adipose tissues. An obese individual is at risk of developing other complications such as diabetes and cancer. Adipogenesis and lipogenesis lead to lipid accumulation due to the differentiation and proliferation of premature adipocytes. These processes are tightly regulated by signaling molecules such as PPARγ, C/EBPα, and SREBP. Therefore, adipogenesis and lipogenesis can be inhibited by suppressing the expression of these key signaling molecules. Obesity can also be treated by suppressing angiogenesis, as it is responsible for nourishing adipose tissues. Although angiogenesis is a normal physiological process, it may lead to the pathogenesis of several diseases by supplying nutrients to target tissues. It promotes adipocyte proliferation, tumor metastasis, and rheumatoid arthritis [43]. It is well-known that angiogenesis promotes the growth of plastic and non-plastic tissues [44]. Adipogenesis and neovascularization are interconnected processes [45].

Natural anti-obesity products are gaining popularity. However, only a few natural products are reported to have anti-obesity effects. They have been proved to be safe and effective for the treatment of obesity; they act by regulating fatty acid oxidation, lipogenesis, and adipogenesis [46]. Microalgae are of great importance and commercial interest because they contain a wide range of biomolecules and active metabolites [47]. Among the various biomolecules reported in the crude extracts of microalgae are phenolic compounds, carotenoids, polyunsaturated fatty acids, and polysaccharides [48, 49]. In the present study, a crude microcystin extract was isolated from *M. aeruginosa* and evaluated for its inhibitory effects on lipid accumulation. The results confirmed that the extract potentially prevents obesity by controlling the proliferation of premature 3 T3 adipocytes. It exerted anti-adipogenic effects in 3 T3-L1 cells in a dose-dependent manner. RT- PCR analysis showed that the extract downregulates lipogenesis- and adipogenesis-related signaling molecules such as PPARγ, C/EBPα, and SREBP. The results of Oil Red O staining also verified that the crude microcystins extract suppresses lipid accumulation in 3 T3-L1 cells in a dose-dependent manner.

## Conclusion

*M. aeruginosa* produce toxic secondary metabolites called microcystins under stress conditions. We proved experimentally that these bioactive toxic metabolites have anti-angiogenic and anti-obesity activities. The results demonstrated that crude microcystin significantly reduce the proliferation and tubes formation of HUVECs and inhibit lipid accumulation in 3 T3-L1 mouse cells line. The RT-PCR analysis showed that the anti-angiogenesis and adipogenesis activities of the microcystin are due to the inhibitory effects on the expression of signal regulator molecules of angiogenesis and adipogenesis. Hence microcystins are crucial in treatment of obesity and tumors. Further studies are continued to identify the pure compounds and their safe utilization in pure form or in pharmaceuticals formulation for obesity and tumors treatment.

## Abbreviations

ANOVA: One-way analysis of variance
C/EBPα: CCAAT/enhancer-binding protein α
DMEM: Dulbecco’s modified eagle’s medium
DMSO: Dimethyl sulfoxide
FBS: Fetal bovine serum
HUVECs: Human umbilical vein endothelial cells
MTT assay: Methylthiazolyl tetrazolium assay
OD: Optical density
SEM: Standard error of the mean
SREBP-1c: Sterol regulatory element binding protein-1c
